# Nicotinamide Mononucleotide improves oocyte maturation of mice with type 1 diabetes

**DOI:** 10.1038/s41387-024-00280-8

**Published:** 2024-04-23

**Authors:** Fucheng Guo, Luyao Wang, Yurong Chen, Haibo Zhu, Xiangpeng Dai, Xiaoling Zhang

**Affiliations:** 1 Key Laboratory of Organ Regeneration and Transplantation of Ministry of Education, First Hospital of Jilin University, Changchun, China; 2 National-Local Joint Engineering Laboratory of Animal Models for Human Disease, First Hospital of Jilin University, Changchun, China; 3 Center of Reproductive Medicine & Center of Prenatal Diagnosis, First Hospital of Jilin University, Changchun, China

**Keywords:** Type 1 diabetes, Type 1 diabetes

## Abstract

**Background:**

The number of patients with type 1 diabetes rises rapidly around the world in recent years. Maternal diabetes has a detrimental effect on reproductive outcomes due to decreased oocyte quality. However, the strategies to improve the oocyte quality and artificial reproductive technology (ART) efficiency of infertile females suffering from diabetes have not been fully studied. In this study, we aimed to examine the effects of nicotinamide mononucleotide (NMN) on oocyte maturation of mouse with type 1 diabetes mouse and explore the underlying mechanisms of NMN’s effect.

**Methods:**

Streptozotocin (STZ) was used to establish the mouse models with type 1 diabetes. The successful establishment of the models was confirmed by the results of body weight test, fasting blood glucose test and haematoxylin and eosin (H&E) staining. The in vitro maturation (IVM) rate of oocytes from diabetic mice was examined. Immunofluorescence staining (IF) was performed to examine the reactive oxygen species (ROS) level, spindle/chromosome structure, mitochondrial function, actin dynamics, DNA damage and histone modification of oocytes, which are potential factors affecting the oocyte quality. The quantitative reverse transcription PCR (RT-qPCR) was used to detect the mRNA levels of Sod1, Opa1, Mfn2, Drp1, Sirt1 and Sirt3 in oocytes.

**Results:**

The NMN supplementation increased the oocyte maturation rate of the mice with diabetes. Furthermore, NMN supplementation improved the oocyte quality by rescuing the actin dynamics, reversing meiotic defects, improving the mitochondrial function, reducing ROS level, suppressing DNA damage and restoring changes in histone modifications of oocytes collected from the mice with diabetes.

**Conclusion:**

NMN could improve the maturation rate and quality of oocytes in STZ-induced diabetic mice, which provides a significant clue for the treatment of infertility of the patients with diabetes.

## Introduction

Type 1 diabetes (T1D), known as the insulin-dependent diabetes or juvenile diabetes, could lead to long-term complications, and subsequently has a significant impact on psychology and life quality of patients. More importantly, studies have shown that diabetes has a detrimental effect on oocyte maturation, preimplantation embryo development and pregnancy outcome [1]. Oocytes at the meiotic maturation stage are easily damaged under hyperglycemic environment caused by type 1 diabetes due to their susceptibility to hyperglycemic environment [2]. Studies have shown that the ovulation rate of diabetic mice has decreased by about 50%, and the number of ovulated oocytes at metaphase II is dramatically reduced [2]. Therefore, patients with diabetes might have a declined oocyte maturation rate and low-quality oocytes, which may be more severe in the infertile patients with diabetes. It has also been reported that oocytes of the mouse with type 1 diabetes showed high level of ROS, abnormal spindle assembly and chromosome alignment, mitochondrial dysfunction and alterations of histone acetylation [3–5]. In addition, the mitochondrial dysfunction and apoptosis can also be observed in cumulus cells of diabetic mouse [6]. Altogether, these results indicated that diabetic mice have impaired oocyte quality and embryonic development, which leading to poor pregnancy outcomes. However, how to improve the maturation rate and quality of oocytes in diabetic animals has not been fully explored.

In vitro maturation refers to the culture process of immature oocytes to the metaphase II (MII) stage in a suitable medium in vitro [7]. IVM provides a powerful tool to study the mechanisms underlying the oocyte maturation and to obtain enough matured oocytes from some large animals for research, such as porcine. Moreover, compared with conventional controlled ovarian hyperstimulation (COH) cycles, IVM has other advantages, including reduced gonadotropin stimulation, gonadotropin stimulation, and the risk of ovarian hyperstimulation syndrome (OHSS), shorter treatment time, and lower costs [8]. However, the efficiency of IVM is affected by multiple factors including the donor health condition, ovary condition, culture method and others [8], which subsequently affected the overall quality of IVM oocytes and caused abnormal development of embryos derived from the IVM oocytes [9]. Therefore, it is essential to optimize the IVM cultivation system to obtain higher quality matured oocytes for further development of embryos.

It has been reported that high-glucose decreased the extrusion rate of first polar body, which is the most important parameter for oocyte maturation, and changed the DNA methylation levels during the oocytes maturation process, which may be responsible for the high risk of chronic diseases in offspring of the mothers with diabetes [10]. Moreover, the polycystic ovary syndrome (PCOS) is frequently found in women with type 1 diabetes. The exploration of treatment strategies for infertility of the patients with PCOS associated with diabetes is extremely significant for these females. The combination of IVM and intracytoplasmic sperm injection (ICSI) provided an effective method to overcome the challenges of infertility treatment in patients with PCOS associated with diabetes [11]. Therefore, IVM may be a promising method to avoid high glucose caused damage to oocytes and to provide oocytes for ART of patients with diabetes. Notably, compared with the naturally matured oocytes, the maturation rate, quality and developmental potential of IVM oocytes still need to be improved. The increased interval between ovulation and fertilization during IVM decreased the mitochondrial membrane potential, followed by an increase in oxidative stress and ROS production in oocytes [12]. Moreover, oxidative stress can damage mitochondrial function, increase apoptosis, and ROS production, and induce DNA damage. Furthermore, oxidative stress can lead to the lower fertilization rate and lower embryo quality, and thus increase the miscarriage rate [13]. Therefore, overcoming the problems correlated with IVM by optimizing the IVM system is of significant importance. Previous studies have shown that supplementation of antioxidants during the IVM process could improve the oocyte quality and reduce the damage caused by oxidative stress [5, 14, 15]. In line with this finding, we aimed to explore novel strategies to improve the maturation rate and quality of IVM oocytes of patients suffering from diabetes, thereby increase the assisted reproductive efficiency and ultimately improve the reproductive outcomes of these patients.

Nicotinamide mononucleotide (NMN) is a key precursor of nicotinamide adenine dinucleotide (NAD+), which can reverse the adverse impact caused by insufficient NAD+, such as mitochondrial dysfunction, increased reactive oxygen species (ROS) production and DNA damage [16, 17]. Preclinical studies have shown that NMN exhibits pharmacological effects on cardiovascular diseases [18], cerebrovascular diseases [19], metabolic diseases [20] and neurological diseases [21]. NMN has also been reported to protect oocytes from benzyl butyl phthalate (BBP) exposure [22], ethylene glycol butyl ether (EGBE) exposure [23] and heat stress [24]. Moreover, NMN could enhance the developmental capability of vitrified bovine oocytes by decreasing the ROS level, reducing endoplasmic reticulum (ER) stress, improving mitochondrial function, and inhibiting apoptosis [25]. Recently, NMN has been proved to exert the beneficial effects on oocytes of aging mice and the mice with obesity [26–28]. Despite the current progress for NMN in improving the quality of oocytes from aging mice and the mice with obesity, the effect of NMN on the oocyte quality of the mice with type 1 diabetes has not been determined.

Therefore, in the study, the effect of NMN on the diabetic oocytes in terms of the in vitro maturation rate and oocyte quality was fully investigated. In details, we firstly established the mouse models with diabetes via intraperitoneal injection of streptozotocin (STZ). The mice with diabetes induced by STZ were further used to investigate the effect of NMN on oocyte quality. Our results showed that the supplementation of NMN improves in vitro maturation percentage of oocytes and oocyte quality from female mice with diabetes partially through recovering mitochondrial function and actin dynamics, reducing oxidative stress, and altering the histone modifications of oocytes.

## Materials and methods

### Generation of diabetic mice

Female ICR mice (five-week-old) were purchased from Charles River Laboratory (Beijing, China). Mice were kept in specific pathogen-free (SPF) conditions on a 12-h light:12-h dark cycle at constant temperature. The female mice were randomly divided into two groups: the T1D group mice were fasted for 5 h and intraperitoneally injected with streptozotocin (200 mg/kg) dissolved in sodium citrate buffer, the control group mice were fasted for 5 h and intraperitoneally injected with of sodium citrate buffer. The mice were tested for fasting blood glucose on the 10th day after injection. The mice with fasting blood glucose (FGB) value higher than 16.7 mmol/L were regarded as successfully established T1D mice models.

### Histological analysis of pancreas

As pancreas are the mainly affected organ for the T1D mice, pancreas was used for histological analysis. The pancreatic tissues of normal mice and T1D mice were harvested and fixed in Bouin’s fixative for at least 24 h. Then the tissue was dehydrated and embedded in paraffin. Paraffin-embedded pancreatic tissue was sectioned at a thickness of 5 μm and further stained with hematoxylin and eosin for histological analysis.

### Oocyte collection and culture

To obtain GV oocytes, cumulus-oocyte complexes (COCs) were isolated from ovaries using syringe needle in M2 medium (Sigma Aldrich, USA). Cumulus cells were removed by pipetting and the fully-grown GV oocytes were collected. For in vitro maturation, GV oocytes were then cultured in M16 medium covered mineral oil (Sigma Aldrich, USA) for 14–16 h at 37 °C, 5% CO_2_. To collect MII oocytes, mice were intraperitoneally injected with 7.5 IU of pregnant horse serum gonadotropin (PMSG) (Ningbo Second Hormone Factory, China) and 7.5 IU of human chorionic gonadotropin (hCG) (Ningbo Second Hormone Factory, China) after 48 h. The COCs were then collected from the enlarged portion of oviducts 13–16 h after hCG injection and cumulus cells were removed with 1 mg/ml hyaluronidase (Sigma Aldrich, USA).

### NMN treatment

According to our primary experimental result, NMN was diluted into the final concentration of 1 μM using M16 medium. Oocytes were divided into Control group, Diabetic group and Dia + NMN group. Oocytes isolated from normal mice and cultured in M16 medium were defined as Control group. Oocytes isolated from diabetic mice and cultured in M16 medium were defined as Diabetic group. Oocytes isolated from diabetic mice and cultured in M16 medium supplemented with NMN were defined as Dia + NMN group. The oocytes of Dia + NMN group were treated with NMN for 14–16 h.

### Quantitative RT-PCR

In vitro maturated MII stage oocytes were used for qRT-PCR analysis. Total RNA was isolated from 50 oocytes and reversed to cDNA using SuperScript™ IV CellsDirect™ cDNA Synthesis Kit (Invitrogen, USA). The cDNA was then quantified by PowerUp SYBR Green Master Mix (Applied Biosystems, USA) using StepOnePlus™ Real-Time PCR System (Applied Biosystems, USA). The primer sequences were listed in Table 1.

**Table 1:** Primer sequences of genes for quantitative real-time PCR.

Gene	Primer sequence
Sirt1	forward, 5’-ATGACGCTGTGGCAGATTGTT-3’
	reverse, 5’-CCGCAAGGCGAGCATAGAT-3’
Sirt3	forward, 5’-CTACATGCACGGTCTGTCGAA-3’
	reverse, 5’-GCCAAAGCGAAGTCAGCCATA-3’
Drp1	forward, 5’-CAGGTGGTGGGATTGGAGAC-3’
	reverse, 5’-CTGGCATAATTGGAATTGGTTT-3’
Opa1	forward, 5’-CCGAGGATAGCTTGAGGGTT-3’
	reverse, 5’-CGTTCTTGGTTTCGTTGTGA-3’
Mfn2	forward, 5’-TTCTTGTGGTCGGAGGAGTG-3’
	reverse, 5’-CTTTGGTGGTCCAGGTCAGT-3’
Sod1	forward, 5’-AACCAGTTGTGTTGTCAGGAC-3’
	reverse, 5’-CCACCATGTTTCTTAGAGTGAGG-3’
actin	forward, 5’-TTCAACACCCCAGCCATG-3’
	reverse, 5’-CCTCGTAGATGGGCACAGT-3’

### Immunofluorescence staining

For immunofluorescence staining, oocytes were fixed in 4% paraformaldehyde for overnight. After three times of wash using phosphate-buffered saline (PBS) with 0.1% polyvinylpyrrolidone (PVP), oocytes were permeabilized in 0.5% Triton X-100 for 20 min at room temperature. Then oocytes were washed three times using PBS with 0.1% PVP and blocked with PBS containing 1% bovine serum albumin (BSA) for 1 h. Then oocytes were incubated with anti-γ-H2A.X antibody (1:100, 9718T, CST), anti-Tubulin antibody (1:100, AF7010, Affinity), anti-H4K16ac antibody (1:200, ab109463, abcam), anti-H3K4me2 antibody (1:200, ab7766, abcam) or anti-H3K4me3 antibody (1:300, ab8580, abcam) at 4 °C for overnight. After three washes using PBS with 0.1% PVP, oocytes were incubated with secondary antibodies for 1 h at 37 °C. Then oocytes were incubated in Hoechest 33342 (10 μg/ml) for 10 min. After three washes, oocytes were transferred to slides and mounted with prolong Gold Antifade Mountant (Invitrogen, USA).

For ROS staining, after three washes using PBS with 0.1% PVP, oocytes were incubated with the Reactive Oxygen Species Assay Kit (Beyotime Biotechnology, China) for 30 min at 37 °C. The excitation wavelength is 488 nm, while the emission wavelength is 525 nm.

For actin cytoskeleton staining, oocytes were fixed in 4% paraformaldehyde for overnight. After three washes using PBS with 0.1% PVP, oocytes were incubated with Actin-Tracker Red (Beyotime Biotechnology, China) for 30 min at 37 °C.

For active mitochondrion staining, oocytes were incubated with MitoTracker Red CMXRos (Thermo Fisher Scientific, USA) for 30 min at 37 °C. The maximum excitation wavelength is 579 nm, while the maximum emission wavelength is 599 nm.

The oocytes were observed under a Zeiss confocal microscope or Fluorescent Inverted microscope.

### Statistical analysis

Data was analyzed using GraphPad Prism 8 and presented as mean ± SEM. Data was analyzed using t-test or chi-squared test based on GraphPad Prism 8 statistical software. *P* < 0.05 was considered to be significant.

## Results

### Establishment of diabetic mice

**Fig. 1:**
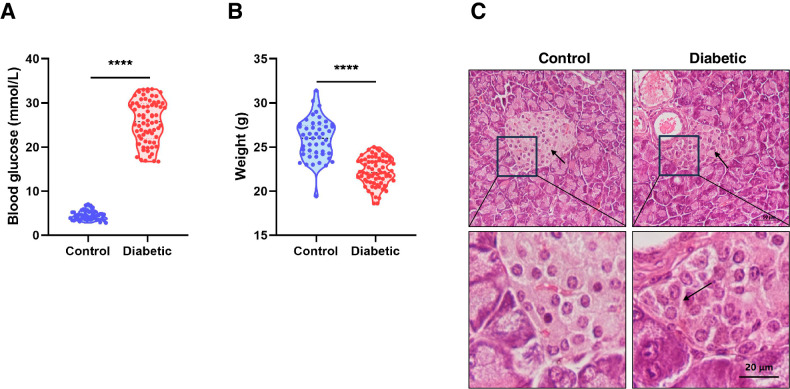
Establishment of the mouse models with type 1 diabetes (T1D). **A** Fasting blood glucose of normal mice (*n* = 49) and the mice with diabetes (*n* = 71). The FGB value of diabetic mice is higher than that of normal mice. **B** The body weight of normal mice (*n* = 49) and the mice with diabetes (*n* = 71). The body weight of the mice with diabetes is lower than that of normal mice. **C** Representative images of H&E staining on pancreatic tissue from normal mice and the mice with diabetes. The morphology of pancreatic islets of control mice was normal, while the pancreatic islets of the mice with diabetes showed decreased volume, β cells aggregation and unclear cell boundaries. Scale bar, 50 μm. ****P < 0.0001.

In our study, the mouse model with type 1 diabetes was established by intraperitoneal injection of STZ solution (200 mg/kg) dissolved in sodium citrate buffer. The body weight and fasting blood glucose of these mice were examined after 10 days. The FGB value of T1D mice (Diabetic) is significantly higher than that of normal mice (Control) (Fig. 1A). However, the body weight of the mice with diabetes is lower than that of normal mice (Fig. 1B). Examination of the H&E sections showed that the morphology of pancreatic islets of control mice was normal, while the pancreatic islets of the diabetic group showed decreased volume, β cells aggregation and unclear cell boundaries (Fig. 1C). These results demonstrated the successful establishment of the mouse models with diabetes.

### NMN improves the reproductive performance of the mice with diabetes

**Fig. 2:**
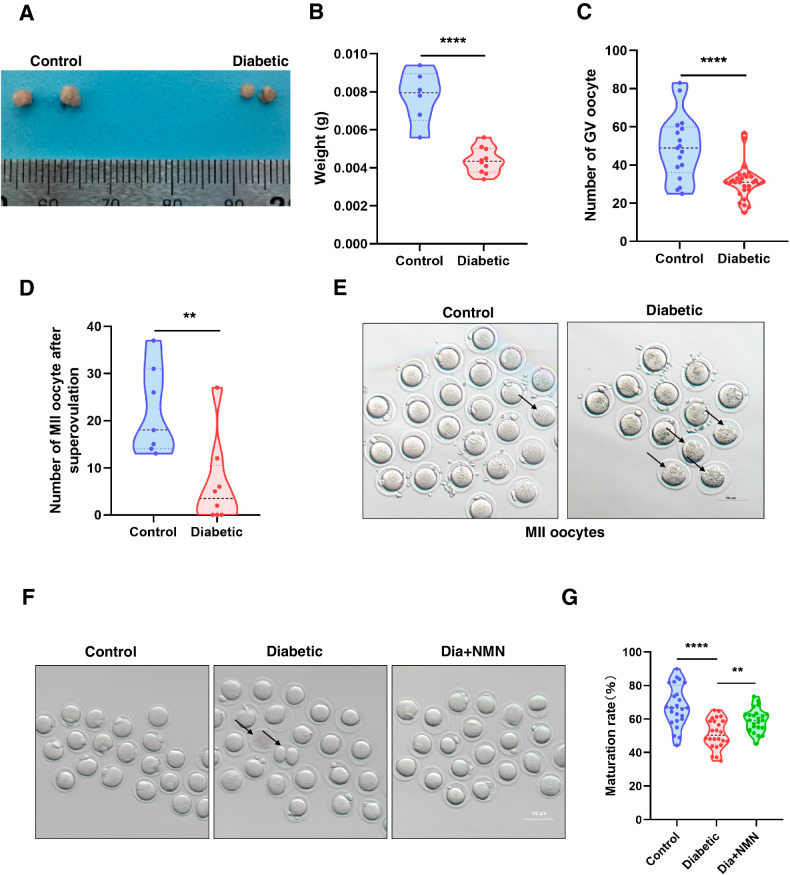
Diabetes negatively affected the reproductive performance of female mice. **A** Representative images of ovaries from normal mice and the mice with diabetes. The ovarian volume of the mice with diabetes was smaller than that of normal mice. **B** The weight of ovaries from normal mice (*n* = 3) and the mice with diabetes (*n* = 5). The ovarian weight of the mice with diabetes was smaller than that of normal mice. **C** The number of GV oocytes collected from normal mice and the mice with diabetes. Compared with that in normal mice, the mice with diabetes have fewer GV oocytes. **D** Number of MII oocytes collected from super-ovulated normal mice and the mice with diabetes. Compared with that in normal mice, the mice with diabetes have fewer MII oocytes. **E** Representative images of MII oocytes collected from normal mice and the mice with diabetes. **F** Representative images of in vitro maturation oocytes in Control, Diabetic, and Dia + NMN group. **G** The in vitro maturation rate of oocytes in Control, Diabetic, and Dia + NMN group. The in vitro maturation rate of GV oocytes in the mice with diabetes was significantly reduced, and NMN supplementation in the culture medium significantly increased the in vitro maturation rate. **P < 0.01; ****P < 0.0001.

Next, we sought to examine the effect of diabetes on reproductive performance of mice. The ovaries were then separated from normal mice and the mice with diabetes. Importantly, compared with the normal mice, both the ovarian volume and ovarian weight of the mice with diabetes were dramatically decreased (Fig. 2A, B). We then collected and counted the GV and MII oocytes from control mice and the mice with diabetes. Notably, the number of GV and MII oocytes in the mice with diabetes were significantly reduced (Fig. 2C–E), which indicated that diabetes plays detrimental effect on mice reproductive performance by dramatically reducing the ovary size and the number of GV oocytes, and the subsequent in vivo matured MII oocytes.

We then sought to examine whether NMN supplementation could improve the oocyte quality of the mice with diabetes. Interestingly, the in vitro maturation rate of oocytes from the mice with diabetes was significantly improved by the NMN supplementation in culture medium (Fig. 2F, G).

### NMN rescues the actin dynamics and reverses meiotic defects in oocytes of the mice with diabetes

**Fig. 3:**
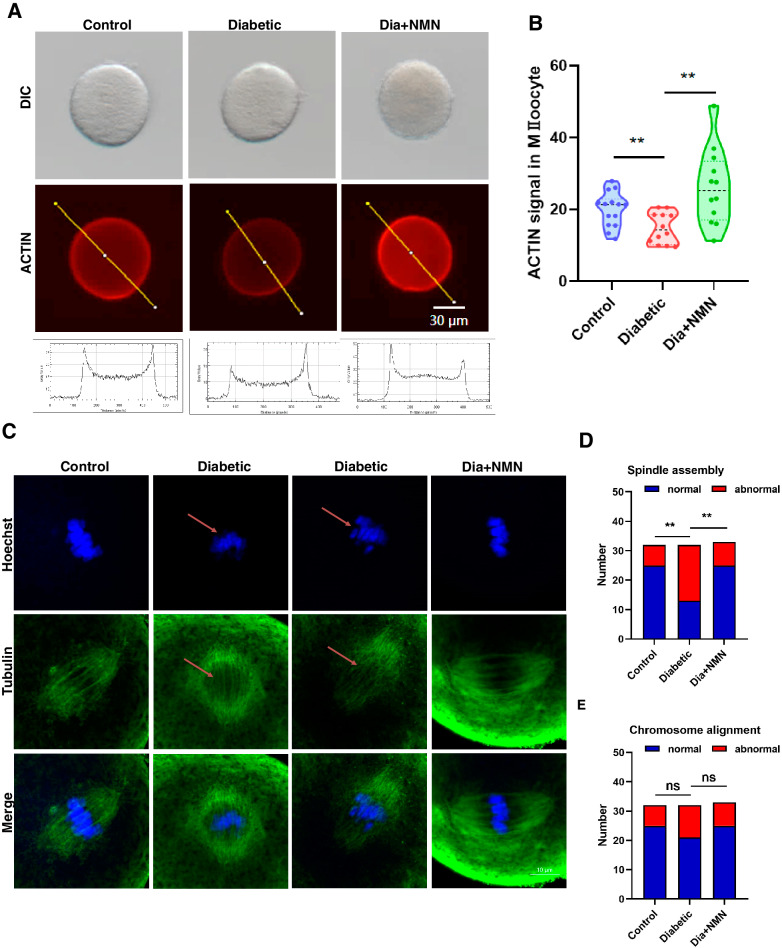
NMN showed positive effect on actin dynamics and spindle/chromosome structure in oocytes of the mice with diabetes. **A** Representative images of actin signals and fluorescence intensity profiling of actin filaments in Control, Diabetic, and Dia + NMN oocytes. Scale bar, 100 μm. **B** The fluorescence intensity of actin filaments on the membrane of Control, Diabetic, and Dia + NMN oocytes was quantified. Compared with normal oocytes, the actin fluorescence intensity on the cytoplasmic membrane of diabetic oocytes was decreased, which could be significantly increased upon NMN treatment (Dia + NMN). **C** Representative images of the spindle morphology and chromosome alignment of in vitro maturated oocytes in Control, Diabetic, and Dia + NMN group. Scale bar, 10 μm. **D** The proportion of normal and abnormal spindle morphology of in vitro maturation oocytes in Control, Diabetic, and Dia + NMN group. The proportion of abnormal spindle morphology of in vitro maturated diabetic oocytes was increased, which could be significantly decreased upon NMN treatment (Dia + NMN). **E** The proportion of normal and abnormal chromosome alignment of in vitro maturated oocytes in Control, Diabetic, and Dia + NMN group. No statistical difference was found between Control, Diabetic, and Dia + NMN group. **P < 0.01.

We then aimed to explore the underlying mechanisms for the improvement of T1D mice oocytes by NMN treatment. The actin cytoskeleton plays an important role in the establishment of cortical polarization and spindle positioning during the oocyte maturation. To test whether the integrity of cytoskeleton of oocytes could be impaired by diabetes, and whether NMN treatment could recover the impaired cytoskeleton integrity of oocytes, we then performed the immunofluorescence (IF) staining to examine the alteration of actin dynamics in oocytes of the mice with diabetes (Fig. 3). The results indicated that, compared with that on the membrane of oocytes from control mice, the actin fluorescence intensity on the oocyte plasma membrane from the mice with diabetes was dramatically reduced. Importantly, the supplementation of NMN could substantially elevate the actin fluorescence intensity on the plasma membrane of the oocytes from the mice with diabetes to a comparative level with oocytes of control mice (Fig. 3A, B). Furthermore, the spindle defects are frequently observed in MII oocytes of the mice with diabetes, while NMN supplementation could markedly reduce the frequency of defect spindles caused by diabetes (Fig. 3C, D). However, statistical difference was not observed in the proportion of chromosomal misalignment among the oocytes of three groups although oocytes from the mice with diabetes showed a slightly increased proportion of abnormal chromosome (Fig. 3E).

### NMN reduces ROS level and improves the mitochondrial function in oocytes of the mice with diabetes

**Fig. 4:**
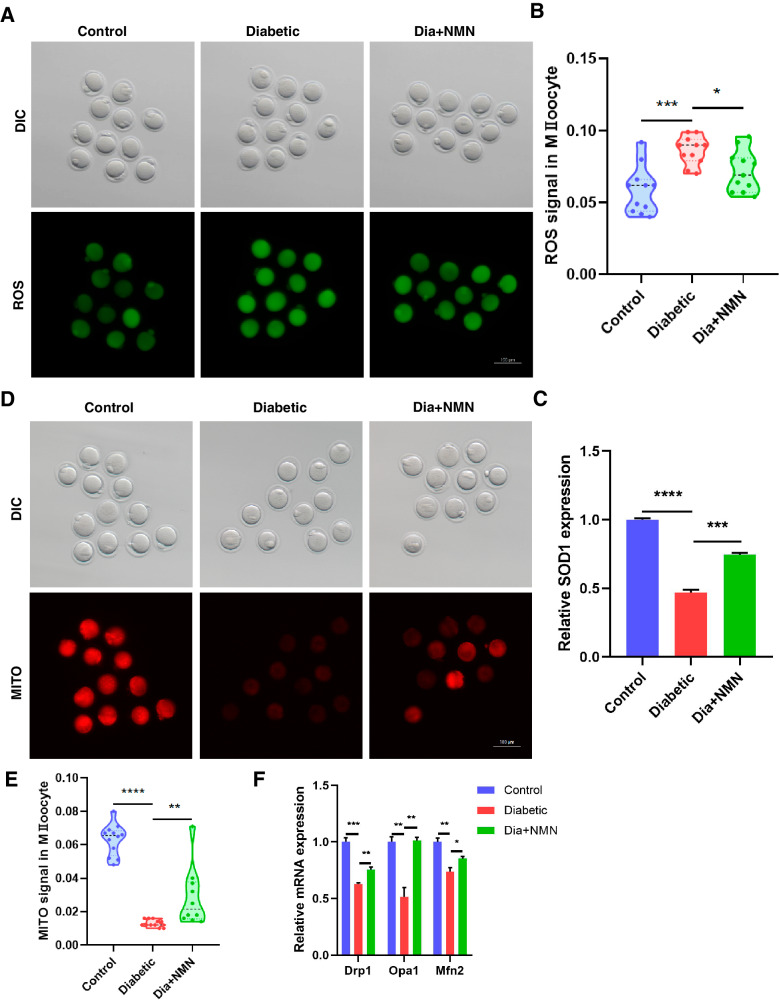
NMN reduced the ROS production and improved the mitochondrial function in oocytes of the mice with diabetes. **A** Representative images of ROS signals in Control, Diabetic, and Dia + NMN oocytes. Scale bar, 100 μm. **B** The fluorescence intensity of ROS in Control, Diabetic, and Dia + NMN oocytes was quantified. The ROS signal of diabetic oocytes was significantly stronger than that in the normal group, while NMN could dramatically reduce ROS level in diabetic oocytes. **C** mRNA level of *Sod1* in Control, Diabetic, and Dia + NMN oocytes was measured by qRT-PCR. The mRNA level of *Sod1* was downregulated in in vitro maturated diabetic oocytes, which could be rescued by NMN treatment (Dia + NMN). The qRT-PCR experiment was repeated for three times. **D** Representative images of mitochondria labeling in Control, Diabetic, and Dia + NMN oocytes. Scale bar, 100 μm. **E** The fluorescence intensity of mitochondrial signals in Control, Diabetic, and Dia + NMN oocytes was quantified. The fluorescence intensity of mitochondria was reduced in diabetic oocytes in comparison with that in the control oocytes, while NMN could effectively elevate the fluorescence intensity of mitochondria in the diabetic oocytes. **F** The mRNA levels of *Drp1*, *Opa1* and *Mfn2* in Control, Diabetic, and Dia + NMN oocytes were measured by qRT-PCR. The mRNA levels of *Drp1*, *Opa1* and *Mfn2* were all downregulated in diabetic oocytes, which could be significantly rescued by the NMN treatment (Dia + NMN). The qRT-PCR experiment was repeated for three times.

It has been reported that the high levels of ROS were frequently detected in the oocytes of T1D mouse, and the increased ROS exhibited detrimental effect on oocyte quality [4]. Therefore, we then examined the ROS level in Control, Diabetic, and Dia+NMN oocytes. Our results indicated that the ROS signal of Diabetic oocytes was significantly stronger than that in the Control oocytes, while NMN could significantly reduce ROS level in Dia+NMN oocytes (Fig. 4A, B). In addition, the results of qRT-PCR showed that the mRNA level of anti-oxidation *Sod1* was downregulated in diabetic oocytes, and it was elevated upon the treatment of NMN (Fig. 4C).

Mitochondria is the energy factory of cells which produces ATP for various cellular events including the cytoskeleton dynamics. Mitochondrial dysfunction is one of the reasons for ROS production. Therefore, we next examined whether mitochondria function is damaged in oocytes of the mice with diabetes and whether NMN could recover mitochondria function. In terms of the mitochondrial labeling, we performed MitoTracker Red staining assay and found that the fluorescence intensity of mitochondria was reduced in diabetic oocytes in comparison with that in the control oocytes, while the fluorescence intensity on mitochondria of the Dia + NMN oocytes was increased (Fig. 4D, E). Furthermore, we then performed RT-qPCR assay to examine the mRNA level of specific genes related to mitochondrial fusion (Opa1 and Mfn2) and mitochondrial fission (Drp1). In line with the results of IF, these genes were all downregulated in diabetic oocytes, while the NMN supplementation could restored these genes to a comparable level with control oocytes (Fig. 4F). These results indicated that the NMN treatment could recover mitochondria function.

### NMN attenuates DNA damage in oocytes of the mice with diabetes

**Fig. 5:**
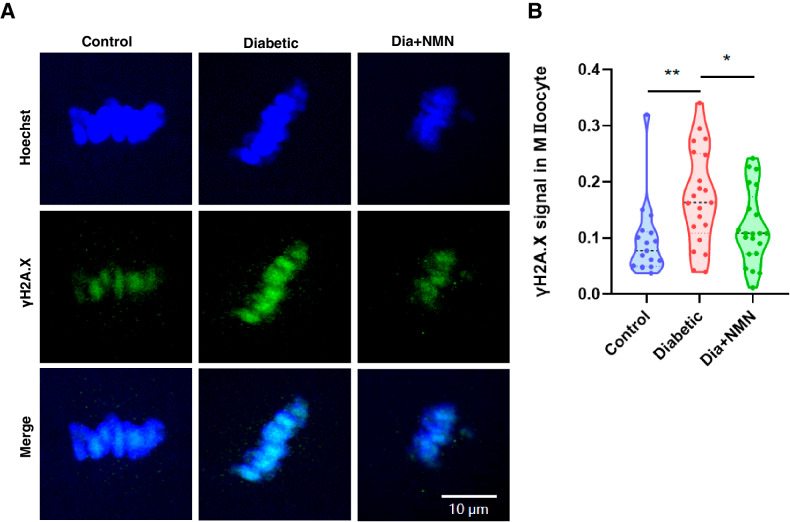
NMN could reduce DNA damage in oocytes from diabetic mice. **A** Representative images of γH2A.X signals in Control, Diabetic, and Dia + NMN oocytes. Scale bar, 10 μm. **B** The fluorescence intensity of γH2A.X signals in Control, Diabetic, and Dia + NMN oocytes was quantified. The fluorescence intensity of γH2A.X signals was elevated in diabetic oocytes in comparison with that in the control oocytes, while NMN could effectively reduce the fluorescence intensity of γH2A.X signals in the diabetic oocytes.

Since high ROS level could induce DNA damage which might affect the oocytes quality, we then assessed the DNA damage accumulation using the γH2A.X antibody. Our results displayed that diabetes could induce a higher percentage of DNA damage, which was reduced by NMN supplementation (Fig. 5A, B).

### NMN regulates the histone modification in oocytes of the mice with diabetes

**Fig. 6:**
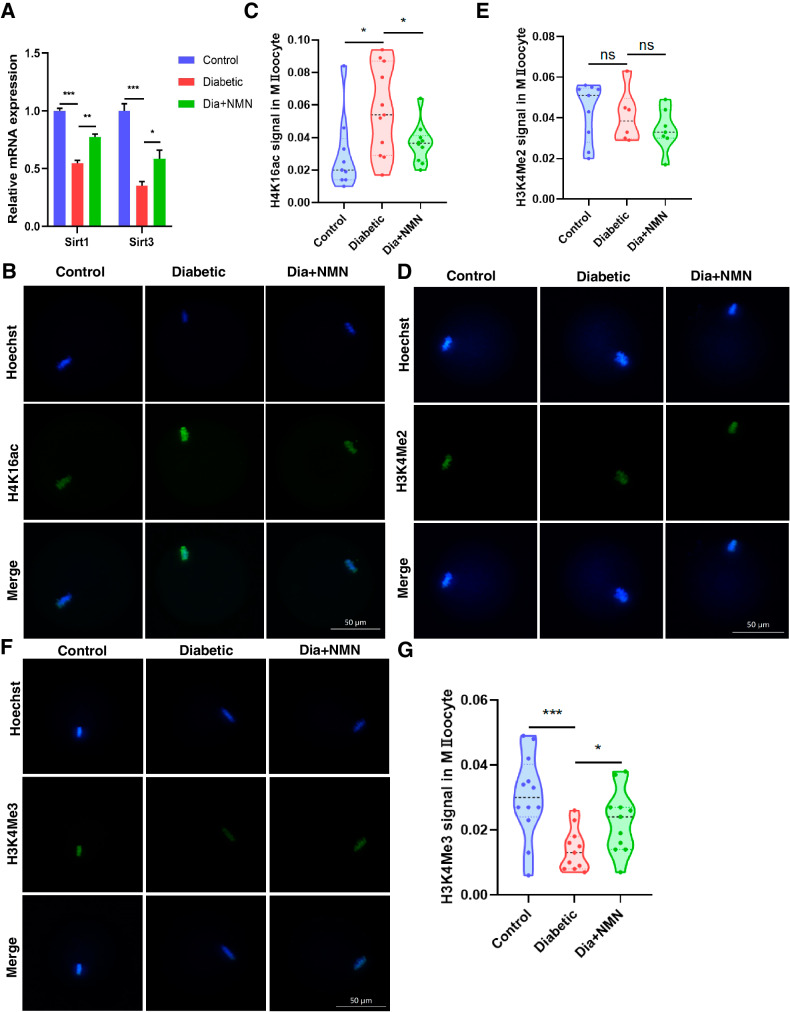
Effect of NMN on H4K16ac, H3K4Me2 and H3K4Me3 in oocytes of the mice with diabetes. **A** The mRNA levels of *Sirt1* and *Sirt3* in Control, Diabetic, and Dia+NMN oocytes were examined by qRT-PCR. The mRNA levels of *Sirt1* and *Sirt3* were decreased in diabetic oocytes, which could be rescued by NMN supplementation (Dia + NMN). The qRT-PCR experiment was repeated for three times. **B** Representative images of H4K16ac signals in Control, Diabetic, and Dia + NMN oocytes. Scale bar, 50 μm. **C** The fluorescence intensity of H4K16ac in Control, Diabetic, and Dia+NMN oocytes was quantified. The H4K16ac signal in diabetic oocytes was significantly higher than that in the control oocytes, and the supplementation of NMN could recover the H4K16ac level of diabetic oocytes. **D** Representative images of H3K4Me2 signals in Control, Diabetic, and Dia + NMN oocytes. Scale bar, 50 μm. **E** The fluorescence intensity of H3K4Me2 in Control, Diabetic, and Dia + NMN oocytes was quantified. There’s no significant difference in H3K4Me2 fluorescence intensity of the oocytes between the three groups. **F** Representative images of H3K4Me3 signals in Control, Diabetic, and Dia + NMN oocytes. Scale bar, 50 μm. **G** The fluorescence intensity of H3K4Me3 in Control, Diabetic, and Dia + NMN oocytes was quantified. The H3K4Me3 signal in diabetic oocytes was significantly lower than that in the control oocytes, and the supplementation of NMN could recover the H3K4Me3 level of diabetic oocytes.

The epigenetic modifications of histone are extremely critical for oocyte maturation and early development of embryos. Notably, the deacetylase SIRT1, as the most prominently studied member of the sirtuins family, plays an important role in preventing apoptosis by deacetylating p53 to inhibit its activity [29]. Previous study indicated that the role of NMN in the recovery of meiotic progression and spindle/chromosome structure of aged oocytes is mediated by Sirt1 [27]. We therefore examined the *Sirt1* expression in Control, Diabetic, and Dia+NMN oocytes. Our results showed that the *Sirt1* mRNA level was decreased in diabetic oocytes, and NMN supplementation could rescue the reduced *Sirt1* expression (Fig. 6A).

Moreover, the mRNA level of the other well studied deacetylase SIRT3 was further tested and the result indicated that, consistent with the reduced expression of *Sirt1*, the mRNA level of *Sirt3* was also significantly decreased in diabetic oocytes, which might be another mechanism for the meiosis disorder and the elevated oxidative stress level in diabetic oocytes [3, 4]. Notably, the supplementation of NMN during in vitro maturation could elevate the mRNA level of *Sirt3* in diabetic oocytes (Fig. 6A).

Given that histone H4K16ac is a target of SIRT1 [30] and histone acetylation and methylation play important roles in oocyte maturation and early embryo development, aberrant histone modifications could impair the developmental competence of oocytes [31]. We next sought to examine their expression in oocytes of three groups. Firstly, we found that the H4K16ac signal in diabetic oocytes was significantly stronger than that in the control group, which is consistent with the result that the expression of *Sirt1* is decreased in diabetic oocytes. Furthermore, the supplementation of NMN could recover the H4K16ac level in diabetic oocytes (Fig. 6B, C).

The histone H3K4me2 and H3K4me3 levels were further examined in the three groups. Interestingly, there’s no significant difference in H3K4me2 fluorescence intensity of the oocytes among the three groups (Fig. 6D, E). However, the H3K4me3 signals were decreased in diabetic oocytes, while NMN supplementation could recover the H3K4me3 signals in diabetic oocytes (Fig. 6F, G).

## Discussion

It was reported that diabetes is harmful to the embryonic development at both pre-implantation stage and post-implantation stage [2, 32]. The mother with diabetes could produce fetuses with higher incidences of malformations and death [33]. Some studies have suggested that the negative effects on embryo development caused by diabetes are associated with the abnormality of oocyte maturation [2–4]. Therefore, how to improve the oocyte quality of infertile women with diabetes to achieve comparative developmental competence with healthy females is an important research and clinical topic. The IVM culture medium plays critical role in achieving higher IVM rate of oocytes [14, 34]. Several drugs have been proved to have the ability to improve oocyte quality when supplemented in the culture medium. For example, the procyanidin B2 demonstrates high efficacy in the improvement of oocyte maturation in the mice with type 1 diabetes [35]. Notably, NMN has been proved to be effective in restoring oocyte quality in aging mice and mice with obesity [26–28]. However, the exact effect of NMN on oocytes of the mice with diabetes has not been fully studied. Here, we found that the number of GV and MII oocytes in the mice with diabetes dramatically reduced, and the ovarian size and ovarian weight of the mice with diabetes are smaller than that of normal mice. Compared with control mice, the polar body extrusion (PBE) rate of IVM oocytes in the mice with diabetes was significantly reduced, indicating that the growth and development ability of oocytes was impaired by diabetic condition. NMN supplementation could improve the in vitro maturation of GV oocytes of the mice with diabetes by rescuing the actin dynamics, reversing meiotic defects, recovering the mitochondrial function, reducing ROS level, suppressing DNA damage and restoring changes of histone modifications in oocytes of the mice with diabetes.

Previous study has shown that the actin dynamics participates in oocyte quality control and NMN could recover the abnormal actin dynamics in oocytes of the mice with obesity and EGBE-exposed porcine oocytes [23, 28]. In line with this notion, our study indicated that diabetes disturbed the integrity of actin in diabetic oocytes and NMN supplementation could recover actin dynamics of in vitro matured oocytes of the mice with diabetes. Moreover, mitochondria play an important role in supplying the energy consumed during oocyte maturation [36, 37]. It has been reported that suboptimal conditions of IVM may lead to mitochondrial dysfunction [38]. In our study, we found that mitochondrial signals declined in in vitro matured oocytes of the mice with diabetes, and NMN treatment could restore the mitochondrial signals. Furthermore, in the process of oocyte maturation, mitochondrial dysfunction hampered its ability to resist the production of ROS, which can further increase the level of oxidative stress [38]. Studies have shown that the ROS level was elevated in oocytes of the mice with diabetes, which could induce DNA damage and apoptosis in oocytes [3, 4, 39]. As NMN has exhibited antioxidant effects in several studies [40–42], we then tested whether the ROS level and DNA damage of diabetic oocytes could be affected by NMN. Interestingly, our results showed that NMN could reduce the ROS level and DNA damage in in vitro matured oocytes of the mice with diabetes.

Mitofusin-2 (MFN2) and Optic atrophy 1 (OPA1) proteins are required for outer and inner mitochondrial membrane fusion, respectively, whereas Dynamin-related protein 1 (DRP1) is the key regulator of mitochondrial fission [43]. MFN2 plays a crucial role in embryo development, and mitochondrial fusion [44]. Importantly, MFN2 is also required for meiotic maturation of mouse oocytes. Research has shown that siRNA mediated knockdown of *Mfn2* in oocytes leads to changes in mitochondrial morphology and quantity which subsequently lead to the abnormality of spindles and chromosomes [44]. OPA1, which exists on the inner mitochondrial membrane (IMM) and is exposed to the intermembrane space (IMS), plays a role in regulating cristae formation by promoting fusion between inner membranes. Dynamin-related protein 1 (DRP1), the member of the dynamin GTPase superfamily, located at the mitochondrial-endoplasmic reticulum interaction site. The main function of DRP1 at the site is regulating mitochondrial division and other cellular processes [45]. Therefore, deficiency of *DRP1* can lead to mitochondrial dysfunction and oxidative stress-induced cell apoptosis during oocyte maturation [45]. Interestingly, our findings revealed that the mRNA levels of genes related to mitochondrial fusion (Opa1, Mfn2) and mitochondrial fission (Drp1) were significantly downregulated in diabetic oocytes, and NMN treatment could restore the mRNA level of these genes in diabetic oocytes. The fact that the mitochondrial fusion and fission related genes in diabetic oocytes were downregulated indicated that the mitochondrial function of oocytes is impaired in the mice with diabetes. NMN treatment could dramatically increase the level of mitochondrial fusion and fission related genes in IVM diabetic oocytes, which proves that NMN can restore the mitochondrial function of in vitro matured diabetic oocytes.

NAD+ plays important role in various biological processes, including metabolism, aging, DNA repair, genomic stability and cell survival [16]. Aberrant NAD+ metabolism has been observed in many aging-associated diseases, such as cancer, cognitive decline, metabolic disease, and others [46]. NMN, the key precursor of NAD+, can enhance NAD+ biosynthesis and reverse defects caused by insufficient NAD+. Study has shown that the age-associated physiological decline in mice which were orally treated by NMN was alleviated, with no obvious toxicity or side effects [47]. The beneficial effects of long-term administration of NMN are manifested in increasing energy metabolism, inhibiting body weight gain during aging, and improving myeloid-lymphoid composition of aged mouse [47]. In the models with diabetes induced by diet or age, NMN treatment could effectively improve impaired glucose tolerance through the enhancement of NAD+ levels, promotion of insulin secretion or insulin sensitivity [20]. Although short-term NMN administration cannot significantly improve fasting blood glucose levels, NMN could effectively restore various metabolic pathways in the models with type 2 diabetes [20]. Studies have shown that NMN exerts its beneficial effect on oocytes of aging mouse and the mouse with obesity through the restoration of NAD+ levels [26, 27]. Moreover, the decline of oocyte quality during aging and metabolic diseases was accompanied with reduced levels of NAD+ [26, 27, 48, 49].

Sirtuins (SIRT1-7) are the NAD+ dependent deacetylases which catalyze protein post-translational modifications [50]. The dysregulation of NAD+ levels can interfere with the deacetylation activity of sirtuins, which can lead to the changes of downstream events, including transcriptional patterns, mitochondrial permeability, mitochondrial ROS (mtROS) production, and oxidative stress [17]. Moreover, inhibition of SIRT1 in mouse oocyte increased ROS levels and abnormal metaphase II plate, proving that SIRT1 might participate in oocyte maturation through the modulation of the redox state and guarantee of normal spindle assembly [51]. SIRT1 was reported to play an important role in regulating the growth and apoptosis of granulosa cells (GCs) [52]. It was found that the ROS content was increased and the *SIRT3* expression was decreased in oocytes of the mouse with obesity [53]. Overexpression of *SIRT3* could reduce ROS accumulation in oocytes of the mouse with obesity [53]. These results demonstrated a critical role of SIRT3 with antioxidant activity in mouse oocytes. Furthermore, study has indicated that incompetent *Sirt3* expression could impair the developmental competence of human IVM oocytes by reducing mitochondrial DNA copy number and biogenesis [54]. As previous study has shown, the decreased expression of *Sirt3* in oocytes of the mice with diabetes may participate in meiosis disorder and ROS accumulation [3, 4]. We then detected the expression of *Sirt3* in oocytes and the results showed that NMN could increase the *Sirt3* expression which is downregulated in IVM oocytes of the mouse with diabetes. Furthermore, NMN may function in the improvement of aged oocytes by elevating the expression of *Sirt1* [27]. Moreover, the antioxidants could protect mice oocytes from postovulatory aging by increasing the *Sirt1* expression [55–57]. Consistently, our results showed that NMN could restore the expression of *Sirt1* in IVM oocytes of the mouse with diabetes. Study has shown that Sirt1 could deacetylate histone H4-K16 [30]. In human cells, RNAi-mediated *Sirt1* knockdown causes hyperacetylation of H4-K16. Our results showed that H4K16ac signals were increased in oocytes of the mouse with diabetes partially due to the decreased expression of *Sirt1*, while NMN supplementation could decrease the H4K16ac signals in oocytes of the mouse with diabetes, which was further confirmed by the fact that the NMN treatment increased expression of *Sirt1*. Abnormal H3K4 methylation has been observed in the maternal aging oocytes and postovulatory aging oocytes [58, 59]. Interestingly, in our study, there was no significant difference of H3K4me2 fluorescence intensity in oocytes among the three groups. In our study, the H3K4me3 signals were decreased in diabetic oocytes, which could be restored by NMN supplementation.

Altogether, in our study, we found that the diabetes exerted detrimental effect on the in vitro maturation of GV oocytes, and NMN might be an effective reagent to improve the quality of in vitro matured oocytes of the mouse with diabetes by restoring mitochondrial function and spindle/chromosome structure, reducing ROS level, rescuing actin dynamics, suppressing DNA damage and restoring histone modifications. Our work provides experiment evidence for the application of NMN in the improvement of the oocyte quality of infertility women with diabetes.

## Data Availability

The datasets generated during and/or analyzed during the current study are available from the corresponding author on reasonable request.
